# Short-Term Seasonal Development of Anthropometry, Body Composition, Physical Fitness, and Sport-Specific Performance in Young Olympic Weightlifters

**DOI:** 10.3390/sports7120242

**Published:** 2019-11-30

**Authors:** Helmi Chaabene, Olaf Prieske, Melanie Lesinski, Ingo Sandau, Urs Granacher

**Affiliations:** 1 Division of Training and Movement Sciences, Research Focus Cognition Sciences, University of Potsdam, Am Neuen Palais 10 (Building 12), D-14469 Potsdam, Germany; chaabanehelmi@hotmail.fr (H.C.); prieske@fhsmp.de (O.P.); mlesinsk@uni-potsdam.de (M.L.); 2 Division of Exercise and Movement, University of Applied Sciences for Sports and Management Potsdam, D-14469 Potsdam, Germany; 3 Research Group Weightlifting, Institute for Applied Training Science, D-04109 Leipzig, Germany; sandau@iat.uni-leipzig.de

**Keywords:** strength, monitoring, young athletes, weight training, somatic variables, periodization, training load

## Abstract

The aim of this study is to monitor short-term seasonal development of young Olympic weightlifters’ anthropometry, body composition, physical fitness, and sport-specific performance. Fifteen male weightlifters aged 13.2 ± 1.3 years participated in this study. Tests for the assessment of anthropometry (e.g., body-height, body-mass), body-composition (e.g., lean-body-mass, relative fat-mass), muscle strength (grip-strength), jump performance (drop-jump (DJ) height, countermovement-jump (CMJ) height, DJ contact time, DJ reactive-strength-index (RSI)), dynamic balance (Y-balance-test), and sport-specific performance (i.e., snatch and clean-and-jerk) were conducted at different time-points (i.e., T1 (baseline), T2 (9 weeks), T3 (20 weeks)). Strength tests (i.e., grip strength, clean-and-jerk and snatch) and training volume were normalized to body mass. Results showed small-to-large increases in body-height, body-mass, lean-body-mass, and lower-limbs lean-mass from T1-to-T2 and T2-to-T3 (∆0.7–6.7%; 0.1 ≤ d ≤ 1.2). For fat-mass, a significant small-sized decrease was found from T1-to-T2 (∆13.1%; d = 0.4) and a significant increase from T2-to-T3 (∆9.1%; d = 0.3). A significant main effect of time was observed for DJ contact time (d = 1.3) with a trend toward a significant decrease from T1-to-T2 (∆–15.3%; d = 0.66; *p* = 0.06). For RSI, significant small increases from T1-to-T2 (∆9.9%, d = 0.5) were noted. Additionally, a significant main effect of time was found for snatch (d = 2.7) and clean-and-jerk (d = 3.1) with significant small-to-moderate increases for both tests from T1-to-T2 and T2-to-T3 (∆4.6–11.3%, d = 0.33 to 0.64). The other tests did not change significantly over time (0.1 ≤ d ≤ 0.8). Results showed significantly higher training volume for sport-specific training during the second period compared with the first period (d = 2.2). Five months of Olympic weightlifting contributed to significant changes in anthropometry, body-composition, and sport-specific performance. However, hardly any significant gains were observed for measures of physical fitness. Coaches are advised to design training programs that target a variety of fitness components to lay an appropriate foundation for later performance as an elite athlete.

## 1. Introduction

Weightlifting has been a longstanding part of the modern Olympic Games with a large and growing international popularity [1]. It is characterized by two technically and physically demanding competitive multi-joint whole body lifts: the snatch as well as the clean and jerk [1,2]. It has been shown that Olympic weightlifting depends upon the capacity of the lifter to accelerate the barbell within a short time-period [3]. Therefore, the ability to produce high peak force and power are important performance determinants in Olympic weightlifting [4,5]. As such, it is not surprising that more successful Olympic weightlifters are stronger and more powerful than their less successful peers [6]. Accordingly, one of the long-term goals with youth Olympic weightlifting is to lay the foundation for high peak power output on the elite level. 

The scientific literature suggests different pathways that may lead to elite performance. These are early specialization (i.e., high volume of sport-specific practice) or early diversification (i.e., variety of sports and activities) [7,8,9]. There is evidence that late specialization is key to success for “centimeters, grams, or seconds sports” (cgs) including weightlifting [9]. In other words, different components of physical fitness should specifically be promoted during the early stages of long-term athlete development while sport-specific skills should be developed during the later stages [10]. In addition, it seems that early specialization is associated with a greater risk of sustaining acute and/or chronic injuries and dropouts [8,11,12,13] while fewer injuries appear to occur with diversification (i.e., late specialization) [13,14]. There is also research available that suggests an increased likelihood of becoming an elite athlete on an international level when following the diversification pathway [9]. 

In the past, these findings have been controversially debated because they are contradictory to more traditional pathways of talent development favoring early specialization [9]. Therefore, the more recent approaches on diversification are far from being acknowledged and implemented in current talent development programs of different sports. An often postulated criticism is that findings supporting diversification pathways are based on retrospective analyses of elite athletes that either made it to a national or international level [15]. Because of the large time lag between the actual analysis and the primary outcome to be evaluated (i.e., long-term athlete development), this retrospective approach is methodologically limited [15]. Therefore, further information is needed on how talent development is being practiced in different sports using different study designs. For this purpose, long-term studies (e.g., single group repeated-measure study designs) should be conducted that monitor physical fitness (e.g., muscle power, force), sport-specific performance (e.g., snatch, clean and jerk), and training data (e.g., training volume, intensity) for certain periods of time during talent development. This is key for the structured development of performance and the prevention of overuse injuries during long-term athlete development. Additionally, this approach should help coaches to evaluate their training on a regular basis by tailoring ongoing decision-making processes [16]. Additionally, it is important from a practitioner’s and researcher’s point of view to systematically monitor anthropometric and body composition data in conjunction with physical performance over the different stages of long-term athlete development, particularly in sports with weight class categories such as weightlifting [1]. In fact, it has previously been shown that weightlifters have shorter arm span and tibial lengths and, accordingly, smaller body heights when compared to other strength and power athletes [17,18]. These anthropometric features may provide biomechanical advantages for lifting heavy loads because joint moments are lower due to shorter resistance lever arms [19]. As such, the total amount of mechanical muscle work is reduced because of shorter vertical lifting distances [19]. 

Further, periodization is a fundamental concept in sports training [20]. The essence of periodization is to find a balance between acute/and chronic training loads and recovery to maximize the training-related adaptations and to reduce the risk of sustaining acute and/or overuse injuries [21]. In general, weightlifting seasons are characterized by long preparation yet short competition periods. This allows to specifically address the different components of physical fitness during long-term athlete development to lay the foundation for high performance as an elite athlete. 

Given the limited information in the scientific literature in young Olympic weightlifters [1], the present exploratory study sought to monitor short-term seasonal development of anthropometry (i.e., body mass, body height), body composition (i.e., lean body mass, and fat mass), physical fitness (i.e., muscle strength, power, and dynamic balance), and sport-specific performance (i.e., snatch and clean and jerk) of young male Olympic weightlifters over a 5 month period. We hypothesized that Olympic weightlifting improves body composition, physical fitness, and sport-specific performance over the course of a 5-month period in young male Olympic weightlifters [22]. 

## 2. Material and Methods

### 2.1. Participants

A total of 15 adolescent male elite weightlifters aged 13.2 ± 1.3 years (maturity offset = +0.61 ± 1.6 years) with an average of 3.5 years of systematic weightlifting training volunteered to participate in this study. Mean body mass and body height amounted to 56.8 ± 16.4 kg and 162.1 ± 11.3 cm, respectively. Participants’ maturity status was estimated using the maturity offset method [23] and the sample included three pre-peak height velocity individuals, seven circa-peak height velocity athletes, and five post-peak height velocity participants. All procedures were approved by the local ethical committee for the use of human participants (University of Potsdam: submission No. 5/2014). The study was conducted in accordance with the latest version of the Declaration of Helsinki. Prior to the start of the study, written informed consent was obtained from the participants and their parents/legal representatives. All participants and their parents/legal representatives were fully informed about the experimental procedures and their potential risks and benefits. Participants were free to withdraw from the study at any time, without providing any reason.

### 2.2. Design and Procedures

**Figure 1:**
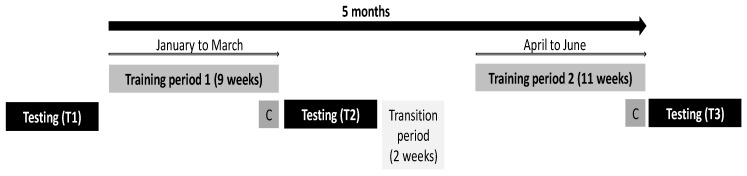
Study design. C: competition.

This is a single group repeated-measure study design conducted to systematically monitor short-term seasonal development of Olympic weightlifters’ anthropometry, body composition, physical fitness and sport-specific performance (i.e., dependent outcome variables) over a 5-months period of Olympic weightlifting training (i.e., the main factor time). The observation period comprised two independent macrocycles. The first macrocycle lasted 9 weeks while the second lasted 11 weeks. Additionally, training data was documented on a regular basis by coaches in an online formular management system (WinWoTa, Institute for Applied Training Science, Leipzig, Germany). Athletes were tested three times over the course of the season (Figure 1). Tests included the assessment of anthropometry, body composition, physical fitness, and sport-specific performance. The anthropometric measures comprised standing and sitting body height, body mass, and body mass index (BMI). The body composition measures involved lean body mass, relative fat mass, and lean upper and lower limb mass. The physical fitness measures included muscle strength (isometric handgrip strength), jump performance (drop-jump (DJ) height, countermovement jump (CMJ) height, DJ contact time, DJ reactive strength index (RSI)), and dynamic balance (Y-balance test). In terms of sport-specific performance, the snatch, as well as the clean and jerk, were conducted. Strength assessment data (i.e., grip strength, clean-and-jerk and snatch) was normalized to body mass. Prior to testing, a standardized warm-up protocol (i.e., 15 min of dynamic stretching, jumping, running and agility/change-of-direction drills) was performed.

### 2.3. Assessment of Anthropometry and Body Composition

In Olympic Weightlifting, measures of body composition such as body mass, lean body mass, and fat mass are essential to be regularly monitored during training [1]. Therefore, body composition (i.e., lean body mass, fat mass, upper/lower limb lean mass) was registered by means of a bioimpedence analysis system (InBody720 system, Biospace, Seoul, Korea). Additionally, standing and sitting height, as well as leg length (i.e., iliac height) were assessed at the beginning of each of the three test sessions using a stadiometer (Seca, Hamburg, Germany). Tests for anthropometry and body composition were always conducted in the morning in a fasting state between 8:00 and 10:00.

### 2.4. Assessment of Physical Fitness 

Isometric handgrip strength was assessed as a general fitness marker and it represents a test for the assessment of maximal muscle strength [24]. Participants sat on a chair with the dynamometer in the dominant hand and the elbow flexed at 90°. The lateral preference inventory questionnaire was used to establish hand dominance [25]. Athletes were asked to press as forcefully as possible with the dominant hand for 3 s. Three trials were carried-out by each participant and the best trial was recorded for further data processing. 

Additionally, jump performance was analyzed to estimate the muscle power of the lower limbs. To assess the jump performance, CMJ and DJ were conducted and measured using an optoelectric device (Optojump, Microgate, Bolzano, Italy). In terms of CMJ performance, athletes started jumping with a countermovement from an upright-standing position, feet shoulder-width apart, and hands akimbo. This was immediately followed by a rapid concentric upward movement. Regarding DJ performance, athletes stepped off a 40-cm box from an upright erect standing position with their dominant leg, feet shoulder-width apart, and hands akimbo, dropped down to land evenly on both feet on the ground, and jumped-off the ground with a double-leg vertical jump at maximal effort. Athletes had to jump as high as possible (CMJ, DJ) in the vertical direction and to keep ground contact time as short as possible (DJ). Proper care was taken to assure a uniform dropping technique for all participants (e.g., extended legs during flight time) [26]. In addition, we monitored that athletes did not jump in forward or backward direction but rather stayed in place to perform their vertical jump. Jump height was calculated using the formula: jump height = 1/8 × g × t², where g is the acceleration due to gravity and t is the flight time [27]. Three CMJs and DJs trials were performed with a rest period of 30 s between the single jump trials and a 1-min rest between the different vertical jump types. The best trial (i.e., maximal jump height) was used for the CMJ. For the DJ, maximal jump height and the best reactive strength index (RSI = ratio of jump height and corresponding ground contact time) trial were taken for further data processing. 

Dynamic balance was assessed by means of the Y-balance-test [28]. Before starting the test, participants’ left and right leg length were assessed. This was done by having athletes in supine lying position. Afterward, the distance from the anterior superior iliac spine to the most distal aspect of the medial malleolus was measured. Further, to have participants acquainted with the testing conditions, three trials per reach direction were practiced on each foot. All trials were conducted barefoot. Participants were positioned in single-leg stance while reaching as far as possible with the contralateral leg in three different reach directions (i.e., anterior, posteromedial, posterolateral). The start of the test was standardized by using the right foot placed at the center of the Y-balance-test tool (Move2Perform, Evansville, IN, USA) and the left leg reaching three times in anterior direction as far as possible. Afterward, the left foot was placed at the center of the grid and the right leg maximally reached in anterior direction. Thereafter, the same test procedure was followed for the remaining reach directions (i.e., posteromedial and posterolateral positioned 135° from the anterior scale). The tester manually measured the distance from the scale of the tool. A composite score was calculated and taken as dependent variable for further data analyses using the following formula: composite score = ((maximum anterior reach distance + maximum posteromedial reach distance + maximum posterolateral reach distance)/(leg length × 3)) × 100 [28].

### 2.5. Assessment of Sport-Specific Performance

Sport-specific performance was assessed by means of the snatch and clean-and-jerk exercises. During the starting position of the snatch, hips, knees, and ankles are flexed. The back is fully extended and the arms are straight [29,30]. When in starting position, begin the snatch with the first pull. This phase is terminated as soon as the barbell reaches the knee level [31]. Following the first pull, the transition phase starts with a realignment of the lifters body to reach the power position which is characterized by slightly flexed knees. This position enables the lifter to optimally accelerate the barbell during the second pull [29]. The goal of the second pull is to maximize barbell speed by fully extending the lower limb joints. This is realized through a well-coordinated and explosive hip and knee extension with plantar flexion. These preparatory actions allow to lift the barbell overhead [29]. In the turnover phase, athletes move the body (i.e., center of mass) rapidly in a downward direction to catch the barbell (catch position). The catch position is characterized by fully extended arms and a deep squat position. In this phase, the athlete decelerates the falling bar while in a deep overhead squat position. From the deep squat position, the athlete finally stands up into an upright position (i.e., recovery phase) [29,30]. In this study, the highest load to successfully complete three repetitions (3-repetition maximum) was used for further analysis.

Further, the 3-repetition maximum was determined for clean-and-jerk performance. As with the snatch, the clean-and-jerk begins with the same starting position and the movement is initiated with the first pull until the barbell reaches the knee level. The transition phase follows after the first pull until the power position is reached. The second pull is characterized by a rapid extension of the knees, hips, and ankles to maximally accelerate barbell velocity. During turnover, elbows are forcefully rotated underneath the barbell and the athlete “jumps” into the front rack position. During the catch phase, participants decelerate the falling bar by adopting a deep squat position with the barbell racked on the front of the shoulders. This is followed by a full leg extension to reach an upright standing position (i.e., recovery phase). In this position, the athlete prepares for the jerk. The jerk starts with the dip phase. Lifters descend into a quarter squat by having the barbell racked on the anterior part of the shoulders. Upon reach of the bottom point of the dip, the athlete begins the thrust by forcefully extending the ankle, knee, and hip joints. As the bar reaches its maximal vertical velocity, the athlete moves under the bar to adopt a split jerk position and to lift the bar overhead (i.e., turnover). Finally, the athlete decelerates the barbell during the catch phase to hold it overhead in split jerk position. Once the bar reaches the overhead position, the recovery from the split jerk position into an upright parallel stand is initiated to finish the lift (i.e., recovery phase).

### 2.6. Monitoring of Training Data

Team coaches tracked day-to-day training data (i.e., exercises, repetitions, loads) for each athlete and each training session over the entire 5-months observation period using an online formular management system (WinWoTa, Institute for Applied Training Science, Leipzig, Germany). The training volume was 4.6 ± 1.3 hours/week. For each macrocycle (i.e., T1–T2; T2–T3), training was coded as sport-specific (e.g., snatch, power cleans, jerks) and non-specific strength training (e.g., dips, bench press, squats) and the respective volume (i.e., tonnage) was calculated as the number of sets × number of repetitions × lifted weight (kg) [32]. Training volume across the two training periods was normalized to body mass.

### 2.7. Statistical Analyses

All values are expressed as means and standard deviations (SD) after normal distribution of data was confirmed through the Shapiro–Wilk test. In order to control for maturity level, an analysis of covariance (ANCOVA) with maturity level as a covariate was used to examine differences in anthropometry, body composition, physical fitness, and sport-specific performance over time. If maturity level failed to be significant, an additional repeated measures analysis of variance (ANOVA) on the factor time was calculated. If a significant effect for the factor time was detected, Bonferroni adjusted post-hoc analyses were applied. Further, training data (i.e., training volume) were analyzed using a two factor repeated measures ANOVA on repeated measures training period (i.e., T1–T2 (macrocycle 1), T2–T3 (macrocycle 2)) and training type (i.e., sport-specific or non-specific training). If interaction effects turned out to be significant, single repeated measures post-hoc ANOVAs were calculated on the factor period for each training type. Effect sizes were calculated by converting partial eta-squared to Cohen’s d. The magnitude of effect sizes was classified as small (0.2 ≤ d < 0.5), medium (0.5 ≤ d < 0.8), and large (d ≥ 0.8) [33]. The α level of significance was set a priori at *p* < 0.05. All analyses were performed using Statistical Package for Social Sciences (SPSS, IBM, Endicott, NY, USA) version 25.0.

## 3. Results

Two participants left the Olympic training center for personal reasons. Accordingly, they were excluded from the study. All participants practiced weightlifting for 4.6 ± 1.2 sessions per week and regularly competed on a national level. 

### 3.1. Anthropometry and Body Composition

Table 1 displays changes in anthropometry and body composition over the course of the 5-months period. Our results showed a significant main effect of time (1.6 ≤ d ≤ 5.7, *p* < 0.05) for most of the variables, except BMI and upper limb lean mass. Post-hoc analyses indicated significant small-to-large increases in body height, body mass, lean body mass, and lower limb lean mass from T1 to T2 (∆0.7–4.4%; 0.1 ≤ d ≤ 0.8, *p* < 0.05), T1 to T3 (∆1.8–6.7%; 0.2 ≤ d ≤ 1.3, *p* < 0.05), and T2 to T3 (∆1.1–3.9%; 0.1 ≤ d ≤ 0.8, *p* < 0.05). In terms of fat mass, results showed a significant small decrease from T1 to T2 (∆13.1%; d = 0.4, *p* < 0.05) and a non-significant small decrease from T1 to T3 (∆2.8%; d = 0.1, *p* > 0.05). From T2 to T3, a significant small increase in fat mass was observed (∆9.1%; d = 0.3, *p* < 0.05). 

### 3.2. Muscle Strength/Power and Dynamic Balance

Table 2 illustrates development in measures of muscle strength and power over the 5-months observational period. Results showed no significant main effect of time for grip strength, CMJ, DJ, and Y-balance-test for the left and right leg (0.1≤ d ≤ 0.8, *p* > 0.05). However, a significant main effect of time was observed for DJ contact time (d = 1.3) with a trend toward a significant decrease from T1-to-T2 (∆–15.3%; d = 0.66; *p* = 0.06). Also, a significant main effect of time was found for RSI (d = 1.1, *p* < 0.05). RSI showed significant and small increases from T1 to T2 (∆9.9%, d = 0.5, *p* < 0.05). 

### 3.3. Sport-Specific Performance

A significant main effect of time was observed for the snatch (d = 2.7, *p* = 0.00) as well as the clean-and-jerk (d = 3.1, *p* = 0.00) (Table 2). Post-hoc analyses showed significant small and consistent increases from T1 to T2 and from T2 to T3 (∆4.6–11.3%, d = 0.33 to 0.64). 

### 3.4. Training Types and Volume

**Figure 2:**
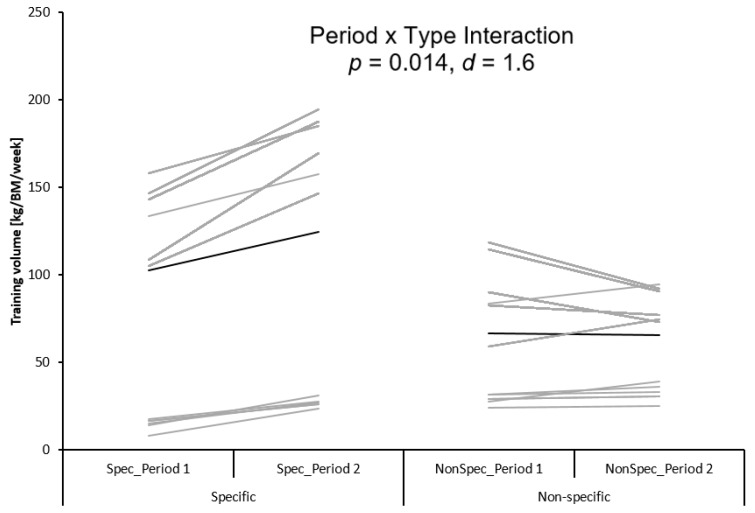
The distribution of specific and non-specific training volume between the first and the second training period. BM = body mass in kg.

The two factor repeated measures ANOVA revealed a significant interaction between training period and training type (d = 1.6, *p* = 0.014). Post-hoc analyses indicated significant increments in training volume for the sport-specific training only from the first (i.e., T1–T2) to the second (i.e., T2–T3) macrocycle (d = 2.2, *p* = 0.001) (Figure 2).

## 4. Discussion

The purpose of this single group repeated-measure study design was to monitor the short-term seasonal development of anthropometry (i.e., body mass, body height, BMI), body composition (i.e., lean body mass, and fat mass), muscle strength, jump performance, dynamic balance, and sport-specific performance (i.e., clean-and-jerk and snatch) over the course of a 5-months period in young male Olympic weightlifters. The main results showed significant, small-to-moderate increases in anthropometry (body height, body mass) and body composition (lean body mass, lower limb lean mass) over the training period. However, hardly any significant gains were observed for measures of physical fitness, except DJ contact time and RSI. In terms of sport-specific performance, our results showed consistent small performance improvements in the snatch as well as the clean-and-jerk over the observation period. 

### 4.1. Anthropometry and Body Composition

Given that weightlifting is a weight category sport, body composition, namely fat mass, and lean body mass, are the two fundamental aspects that need to be regularly monitored during athletic development [1]. Specifically, while fat mass has to be kept low, athletes and coaches strive to increase lean body mass as it is associated with greater strength and power performances [1]. In fact, it has been shown that the total amount of lean body mass influences the level of strength and power across all ages and weight categories in Olympic weightlifting [34,35]. In the same context, lower fat mass was recorded in elite compared with sub-elite weightlifters of similar total body mass [17,36]. All these findings highlight the importance of systematically monitoring anthropometry and body composition, namely fat mass and lean body mass of weightlifters during talent development. 

Our findings showed significant small-to-moderate increases in body height, body mass, lean body mass, and lower limb lean mass over the course of the 5-months period. In terms of fat mass, a significant and small decrease was observed from T1 to T2. To the best of our knowledge, there is only one study that monitored changes in anthropometry and body composition over a 5-months training period in adult but not youth male weightlifters aged 28 years [22]. These authors reported a small increase (<2%) in body mass over the course of training. Yet, larger variations have been shown for fat-free mass (∆+3.6%) and fat mass (∆−7.1%) [22]. 

It is well-documented that changes in anthropometrics and body composition are highly affected by the individual’s level of maturation [37]. In accordance with Armstrong and McManus [38], the male adolescent growth spurt predominately covers a period of ~4 years around peak height velocity with peak height velocity being reached between 13.4 and 14.4 years. At baseline our participants were classified as pre- peak height velocity (n = 3), around- peak height velocity (n = 7), and post- peak height velocity (n = 5) with an overall mean age of 13.2 years. Thus, it can be hypothesized that growth and maturation partly contributed to the observed changes in anthropometry and/or body composition. For instance, the observed changes in body height of ⁓3 cm are well in-line with the body height increases reported for German adolescent males with a mean age of 13 years over a similar time period [39]. Consequently, the changes in anthropometry/body composition are multifactorial and can most likely be attributed to the combined effects of training, diet, growth, and/or maturation.

### 4.2. Muscle Strength/Power, Dynamic Balance, and Sport-Specific Performance

Weightlifting is characterized by two main whole-body lifts. The snatch as well as the clean-and-jerk. These lifts are technically complex and afford a series of high-intensity muscular actions [2]. The main goal of weightlifters is to be able to sufficiently accelerate the barbell to lift more than their opponents [2]. Accordingly, weightlifters have to generate high peak force and power during sport-specific drills [5,40]. In fact, it has been shown that more successful weightlifters acquire higher power performance compared with their less successful peers [6]. 

Our findings showed that hardly any significant gains were observed for measures of muscle strength, jump performance, and dynamic balance, except DJ contact time and RSI. However, sport-specific performance (i.e., clean-and-jerk and snatch) displayed significant and consistent increases across the overall training period. The mean increase of 16 kg total (sum of snatch and clean-and-jerk) over 5 months is similar to youth male weightlifter of the same age in the study of Byrd et al. [41]. The consistent increase in sport-specific performance could be due to the increased sport-specific training load, better technical skill competency, and/or maturation. We observed significant increases in the total training volume of the sport-specific training (e.g., snatch, power cleans, jerks) from the first (i.e., T1–T2) to the second (i.e., T2–T3) macrocycle. Accordingly, the observed sport-specific performance gains from T1 to T3 in the snatch, as well as the clean-and-jerk, could be due to increased volume of sport-specific training over the macrocycles [42,43]. However, whether the early involvement in the main sport (i.e., early specialization) will be safe and allow further performance improvements in later stages of long-term athlete development still an open question that needs to be addressed in future long-term studies. However, it has frequently been reported that early specialization may lead to negative consequences at later stages of long-term athlete development (e.g., higher dropouts and risk of injuries) [8,9,10,11,12,13,44]. Accordingly, this pathway was deemed potentially detrimental when adopted at early stages of long-term athlete development. Alternatively, late specialization with reduced levels of sport-specific training was recommended [9,10,44]. In fact, balanced and diversified development of physical fitness at early long-term athlete development stages appears vital for a successful career as an elite athlete in cgs sports [7,9,45]. Therefore, coaches are advised to follow an early diversification approach by targeting the development of a wide range of physical fitness components. Such an approach contributes establishing an appropriate foundation for subsequent performance as an elite athlete, thereby increasing the likelihood of achieving long-term athletic success [8]. In general, studies addressing the chronic effects of weightlifting training on physical fitness measures in young weightlifters are scarce [1]. The few available studies examined short-term (i.e., 8 to 10 weeks) effects of weightlifting training on physical fitness (e.g., muscle strength and power) [46,47] and neuromuscular adaptations in weightlifters [48]. To the authors’ knowledge, there is only one study available [22] that examined the effects of a five months weightlifting training on strength and power performance. However, this study included a small and heterogeneous sample (i.e., seven individuals including three females) of elite weightlifters aged 28 years (males) and 23 years (females). These authors [22] showed substantial changes in maximal muscle strength (i.e., isometric mid-thigh clean pull) and power (i.e., loaded vertical jumping) after training. Additionally, they revealed significant large-to-nearly perfect associations (Pearson’s r = 0.62 to 0.98) between weightlifting total performances as well as strength and power measures. These findings indicate that stronger and more powerful weightlifters are those that can generate better weightlifting performance [22]. Overall, there is compelling evidence that the development of a foundation of physical fitness qualities during the early long-term athlete development stages is better than an early focus on sport-specific training [10]. Late compared with early specialization is associated with lower injury prevalence rates and less dropouts from sports. Also, late specialization contributes to build the foundation on which more advanced and complex training can be later developed [9,10]. Therefore, practitioners and coaches are advised to design training interventions that target various physical fitness components during the early long-term athlete development stages [10]. 

### 4.3. Limitations

This study does have some limitations that need to be discussed. First, the maturity-related changes in performance is a confounding factor that could have affected variations in anthropometry/body composition, muscle strength/power and sport-specific performance in our sample. However, the applied statistical approach allowed us to control for differences in maturity level. Second, the sample size included in this study is rather small. However, the size of the overall population of youth Olympic weightlifters is small compared with other sports (e.g., soccer) which consequently results in a limited sample size. The included young athletes were recruited from a large Olympic testing and training center representing German high-level young weightlifters. Third, jump height was not directly measured but estimated from flight time. However, we closely monitored the jump execution to avoid bias from flight time assessment. Moreover, we did not include sport-specific strength tests with movement patterns similar to Olympic lifts such as front and back squats [1]. This needs to be considered in future research. Finally, we did not test our sample after the two-weeks transition period between the two training cycles. An additional test point right after the transition period could have provided more detailed information on the temporal changes that may have occurred in anthropometry, body composition, physical fitness, and sport-specific performance in young male Olympic weightlifters. 

## 5. Conclusions

The main outcomes of the current study showed significant, small-to-moderate increases in anthropometry (body height, body mass) and body composition (lean body mass, lower limb lean mass) over the course of the 5-months observation period. However, hardly any significant gains were observed for measures of physical fitness. Regarding sport-specific performance, our results showed consistent but small performance improvements in the snatch as well as the clean-and-jerk. With reference to the principle of training specificity, the identified increase in sport-specific training volume over the macrocycles could have contributed to the observed increases in weightlifters’ sport-specific performance. It has frequently been reported that early specialization can have negative effects on young athletes’ development because of the dropouts and/or a high risk of sustaining injuries. Moreover, there is evidence that late specialization can be beneficial for sporting success as an elite athlete. 

## 6. Practical Applications

Practitioners are advised to design training programs that promote various physical fitness qualities during the early long-term athlete development stages. Such an approach was deemed effective to build a solid foundation on which more advanced and complex training can be developed during later long-term athlete development stages. Future long-term studies are needed that contrast effects and implications of sport-specific versus non-specific training during long-term athlete development in youth Olympic weightlifters. 

## Figures and Tables

**Table 1:** Change in anthropometry and body composition in young male elite weightlifters across 5 months of training.

Anthropometric and Body Composition Variables	N	T1 (Mean and SD)	T2 (Mean and SD)	T3 (Mean and SD)	T1–T2 (%)	T1–T3 (%)	T2–T3 (%)	Time (d)
Body height (cm)	13	161.4 ± 11.6	162.6 ± 11.5	164.4 ± 11.1	0.7 **	1.8 ***	1.1 ***	5.7 ***
Body mass (kg)	13	55.5 ± 2.8	56.3 ± 2.8	58.6 ± 3.0	1.5	5.4 **	3.9 **	1.6 *
BMI (kg/m^2^)	13	20.9 ± 0.6	20.9 ± 0.5	21.3 ± 0.6	0.3	2.1	1.8	1.0
Lean body mass (kg)	12	26.2 ± 1.5	27.4 ± 1.5	28.1 ± 1.5	4.4 ***	6.7 ***	2.4 **	1.7 **
Fat mass (%)	12	16.9 ± 5.0	15.0 ± 4.9	16.5 ± 5.0	−13.1 **	−2.8	9.1 **	1.9 **
Upper limb lean mass (kg)	12	4.9 ± 0.3	5.2 ± 0.3	5.6 ± 0.4	5.9	12.3	6.8	0.3
Lower limb lean mass (kg)	12	14.1 ± 0.8	14.5 ± 0.8	15.0 ± 0.8	2.5 **	6.2 ***	3.7 ***	2.2 ***

* *p* < 0.05; ** *p* < 0.01; *** *p* < 0.001; BMI: body mass index, d: Cohen’s d.

**Table 2:** Change in performance measures in young male Olympic weightlifters across 5 months of training.

Measures of Physical Fitness and Sport-Specific Performance	N	T1	T2	T3	T1–T2 (%)	T1–T3 (%)	T2–T3 (%)	Time (d)
		(Mean and SD)	(Mean and SD)	(Mean and SD)				
Grip strength (kg/BM)	13	0.54 ± 0.06	0.57 ± 0.07	0.56 ± 0.07	6.2	4.3	−1.7	0.8
CMJ (cm)	13	28.0 ± 6.2	28.5 ± 5.46	29.1 ± 3.7	3.4	3.9	1.9	0.4
DJ (cm)	13	22.9 ± 3.5	23.9 ± 5.3	23.1 ± 3.8	3.9	0.8	−3.2	0.3
RSI (m/s)	13	1.0 ± 0.2	1.1 ± 0.3	1.0 ± 0.2	9.9 *	4.8	−5.7	1.1 *
DJ contact time (ms)	13	255.1 ± 57.8	216.7 ± 15.0	222.9 ± 28.4	−15.3	−12.6	2.9	1.3
Y–balance–test right leg (score)	13	101.5 ± 5.2	101.0 ± 5.9	101.3 ± 6.2	−0.5	−0.2	0.3	0.1
Y-balance-test left leg (score)	13	101.7 ± 4.8	101.3 ± 7.7	103.6 ± 6.2	−0.4	1.8	2.2	0.7
Snatch (kg/BM)	9	0.79 ± 0.14	0.84 ± 0.12	0.88 ± 0.10	6.2 **	11.3 **	4.8	2.7 ***
Clean-and-Jerk (kg/BM)	9	1.00 ± 0.17	1.05 ± 0.15	1.10 ± 0.15	4.9 *	9.8 **	4.6*	3.1 ***

* *p* < 0.05; ** *p* < 0.01; *** *p* < 0.001; CMJ: countermovement jump; DJ: drop jump; RSI: Reactive strength index; d: Cohen’s d.

## References

[1] Storey A., Smith H.K. (2012). Unique aspects of competitive weightlifting: Performance, training and physiology. Sports Med..

[2] Soriano M.A., Suchomel T.J., Comfort P. (2019). Weightlifting Overhead Pressing Derivatives: A Review of the Literature. Sports Med..

[3] Kauhanen H. (1984). A biomechanical analysis of the snatch and clean & jerk techniques of Finish elite and district level weightlifters. Scand. J. Sports Sci..

[4] Stone M.H., Sands W.A., Pierce K.C., Carlock J., Cardinale M., Newton R.U. (2005). Relationship of maximum strength to weightlifting performance. Med. Sci. Sports Exerc..

[5] Garhammer J. (1985). Biomechanical profiles of Olympic weightlifters. Int. J. Sport Biomech..

[6] Carlock J.M., Smith S.L., Hartman M.J., Morris R.T., Ciroslan D.A., Pierce K.C., Newton R.U., Harman E.A., Sands W.A., Stone M.H. (2004). The relationship between vertical jump power estimates and weightlifting ability: A field-test approach. J. Strength Cond. Res..

[7] Côté J., Vierimaa M. (2014). The developmental model of sport participation: 15 years after its first conceptualization. Sci. Sports.

[8] CôTé J., Lidor R., Hackfort D. (2009). ISSP position stand: To sample or to specialize? Seven postulates about youth sport activities that lead to continued participation and elite performance. Int. J. Sport Exerc. Psychol..

[9] Moesch K., Elbe A.M., Hauge M.L., Wikman J.M. (2011). Late specialization: The key to success in centimeters, grams, or seconds (cgs) sports. Scand. J. Med. Sci. Sports.

[10] Lloyd R.S., Oliver J.L., Faigenbaum A.D., Howard R., De Ste Croix M.B., Williams C.A., Best T.M., Alvar B.A., Micheli L.J., Thomas D.P. (2015). Long-term athletic development—Part 1: A pathway for all youth. J. Strength Cond. Res..

[11] DiFiori J.P., Benjamin H.J., Brenner J.S., Gregory A., Jayanthi N., Landry G.L., Luke A. (2014). Overuse injuries and burnout in youth sports: A position statement from the American Medical Society for Sports Medicine. Br. J. Sports Med..

[12] Post E.G., Trigsted S.M., Riekena J.W., Hetzel S., McGuine T.A., Brooks M.A., Bell D.R. (2017). The Association of Sport Specialization and Training Volume with Injury History in Youth Athletes. Am. J. Sports Med..

[13] Brenner J.S. (2016). Sports Specialization and Intensive Training in Young Athletes. Pediatrics.

[14] Gullich A., Emrich E. (2014). Considering long-term sustainability in the development of world class success. Eur. J. Sport Sci..

[15] Hodges N.J., Huys R., Starkes J.L., Eklund R.C., Tenenbaum G. (2007). Methodological review and evaluation of research in expert performance in sport. Handbook of Sport Psychology.

[16] Bourdon P.C., Cardinale M., Murray A., Gastin P., Kellmann M., Varley M.C., Gabbett T.J., Coutts A.J., Burgess D.J., Gregson W. (2017). Monitoring Athlete Training Loads: Consensus Statement. Int. J. Sports Physiol. Perform..

[17] Fry A.C., Ciroslan D., Fry M.D., LeRoux C.D., Schilling B.K., Chiu L.Z. (2006). Anthropometric and performance variables discriminating elite American junior men weightlifters. J. Strength Cond. Res..

[18] Marchocka M., Smuk E. (1984). Analysis of body build of senior weightlifters with particular regard for proportions. Biol. Sport.

[19] Keogh J.W., Hume P.A., Pearson S.N., Mellow P. (2007). Anthropometric dimensions of male powerlifters of varying body mass. J. Sports Sci..

[20] Haff G.G., Jeffreys I., Moody J. (2016). 17 The essentials of periodisation. Strength and Conditioning for Sports Performance.

[21] Bompa T.O., Haff G. (2009). Periodization: Theory and Methodology of Training.

[22] Hornsby W.G., Gentles J.A., MacDonald C.J., Mizuguchi S., Ramsey M.W., Stone M.H. (2017). Maximum Strength, Rate of Force Development, Jump Height, and Peak Power Alterations in Weightlifters across Five Months of Training. Sports.

[23] Mirwald R.L., Baxter-Jones A.D., Bailey D.A., Beunen G.P. (2002). An assessment of maturity from anthropometric measurements. Med. Sci. Sports Exerc..

[24] Wind A.E., Takken T., Helders P.J., Engelbert R.H. (2010). Is grip strength a predictor for total muscle strength in healthy children, adolescents, and young adults?. Eur. J. Pediatrics.

[25] Coren S. (1993). The lateral preference inventory for measurement of handedness, footedness, eyedness, and earedness: Norms for young adults. Bull. Psychon. Soc..

[26] Kibele A. (1999). Technical note. Possible errors in the comparative evaluation of drop jumps from different heights. Ergonomics.

[27] Prieske O., Muehlbauer T., Krueger T., Kibele A., Behm D., Granacher U. (2015). Sex-specific effects of surface instability on drop jump and landing biomechanics. Int. J. Sports Med..

[28] Filipa A., Byrnes R., Paterno M.V., Myer G.D., Hewett T.E. (2010). Neuromuscular training improves performance on the star excursion balance test in young female athletes. J. Orthop. Sports Phys. Ther..

[29] Stone M.H., Pierce K.C., Sands W.A., Stone M.E. (2006). Weightlifting: A brief overview. Strength Cond. J..

[30] Takano B. (1987). Coaching optimal technique in the snatch and the clean and jerk: Part II. Strength Cond. J..

[31] Hydock D. (2001). The weightlifting pull in power development. Strength Cond. J..

[32] Scott B.R., Duthie G.M., Thornton H.R., Dascombe B.J. (2016). Training Monitoring for Resistance Exercise: Theory and Applications. Sports Med..

[33] Cohen J. (1988). Statistical Power Analysis for the Behavioral Sciences.

[34] The D.J., Ploutz-Snyder L. (2003). Age, body mass, and gender as predictors of masters olympic weightlifting performance. Med. Sci. Sports Exerc..

[35] Ford L.E., Detterline A.J., Ho K.K., Cao W. (2000). Gender- and height-related limits of muscle strength in world weightlifting champions. J. Appl. Physiol..

[36] Katch V.L., Katch F.I., Moffatt R., Gittleson M. (1980). Muscular development and lean body weight in body builders and weight lifters. Med. Sci. Sports Exerc..

[37] Armstrong N., van Mechelen W. (2017). Children’s Sport and Exercise Medicine.

[38] Armstrong N., McManus A.M. (2011). Physiology of elite young male athletes. Med. Sport Sci..

[39] Kromeyer-Hauschild K., Wabitsch M., Kunze D., Geller F., Geiß H.C., Hesse V., von Hippel A., Jaeger U., Johnsen D., Korte W. (2001). Perzentile für den Body-mass-Index für das Kindes- und Jugendalter unter Heranziehung verschiedener deutscher Stichproben. Monatsschrift Kinderheilkunde.

[40] Garhammer J. (1980). Power production by Olympic weightlifters. Med. Sci. Sports Exerc..

[41] Byrd R., Pierce K., Rielly L., Brady J. (2003). Strength and Conditioning (Michael Stone Sub-editor): Young weightlifters’ performance across time. Sports Biomech..

[42] Behm D.G., Sale D.G. (1993). Velocity specificity of resistance training. Sports Med..

[43] Morrissey M.C., Harman E.A., Johnson M.J. (1995). Resistance training modes: Specificity and effectiveness. Med. Sci. Sports Exerc..

[44] Lloyd R.S., Cronin J.B., Faigenbaum A.D., Haff G.G., Howard R., Kraemer W.J., Micheli L.J., Myer G.D., Oliver J.L. (2016). National Strength and Conditioning Association Position Statement on Long-Term Athletic Development. J. Strength Cond. Res..

[45] Granacher U., Lesinski M., Busch D., Muehlbauer T., Prieske O., Puta C., Gollhofer A., Behm D.G. (2016). Effects of Resistance Training in Youth Athletes on Muscular Fitness and Athletic Performance: A Conceptual Model for Long-Term Athlete Development. Front. Physiol..

[46] Teo S.Y., Newton M.J., Newton R.U., Dempsey A.R., Fairchild T.J. (2016). Comparing the Effectiveness of a Short-Term Vertical Jump vs. Weightlifting Program on Athletic Power Development. J. Strength Cond. Res..

[47] Gonzalez-Badillo J.J., Izquierdo M., Gorostiaga E.M. (2006). Moderate volume of high relative training intensity produces greater strength gains compared with low and high volumes in competitive weightlifters. J. Strength Cond. Res..

[48] Arabatzi F., Kellis E. (2012). Olympic weightlifting training causes different knee muscle-coactivation adaptations compared with traditional weight training. J. Strength Cond. Res..

